# Perceptions of the mental health impact of intimate partner violence and health service responses in Malawi

**DOI:** 10.3402/gha.v7.24816

**Published:** 2014-09-12

**Authors:** Lignet Chepuka, Miriam Taegtmeyer, Genesis Chorwe-Sungani, Janet Mambulasa, Ellen Chirwa, Rachel Tolhurst

**Affiliations:** 1 Medical and Surgical Nursing department, Kamuzu College of Nursing, Blantyre, Malawi; 2 Department of International Public Health, Liverpool School of Tropical Medicine, Liverpool, UK

**Keywords:** health care providers, intimate partner violence, Malawi, mental health, counselling, GBV services

## Abstract

**Background and objectives:**

This study explores the perceptions of a wide range of stakeholders in Malawi towards the mental health impact of intimate partner violence (IPV) and the capacity of health services for addressing these.

**Design:**

In-depth interviews (IDIs) and focus group discussions (FGDs) were conducted in three areas of Blantyre district, and in two additional districts. A total of 10 FGDs, 1 small group, and 14 IDIs with health care providers; 18 FGDs and 1 small group with male and female, urban and rural community members; 7 IDIs with female survivors; and 26 key informant interviews and 1 small group with government ministry staff, donors, gender-based violence service providers, religious institutions, and police were conducted. A thematic framework analysis method was applied to emerging themes.

**Results:**

The significant mental health impact of IPV was mentioned by all participants and formal care seeking was thought to be impeded by social pressures to resolve conflict, and fear of judgemental attitudes. Providers felt inadequately prepared to handle the psychosocial and mental health consequences of IPV; this was complicated by staff shortages, a lack of clarity on the mandate of the health sector, as well as confusion over the definition and need for ‘counselling’. Referral options to other sectors for mental health support were perceived as limited but the restructuring of the Ministry of Health to cover violence prevention, mental health, and alcohol and drug misuse under a single unit provides an opportunity.

**Conclusion:**

Despite widespread recognition of the burden of IPV-associated mental health problems in Malawi, there is limited capacity to support affected individuals at community or health sector level. Participants highlighted potential entry points to health services as well as local and national opportunities for interventions that are culturally appropriate and are built on local structures and resilience.

Intimate partner violence (IPV) has been defined as any form of behaviour within an intimate relationship that causes physical (slapping, kicking, hitting or beating), sexual, or psychological harm (intimidation or constant humiliation). It includes acts of physical aggression, psychological abuse, or forced sexual intercourse, or any form of controlling behaviour (isolating the person from family and friends), monitoring their movements and restricting access to information or assistance (1). ‘Intimate relationship’ in this paper refers to dating or pre-marital relationships, cohabiting, or marital relationships. The impact of IPV on mental health and well-being is well documented in many countries (2), including Southern Africa (3, 4). There are a range of presentations, and depression is reported as the most common mental health effect (4–7). Survivors of IPV also have a greater risk of suicide (5). Other conditions include post-traumatic disorder, anxiety, self-harm, sleep disorders, emotional distress, memory loss, poor self-esteem, fears, worries, and poor social relationships (8–11). IPV, poor mental health and its consequences in terms of low self-esteem have been linked to alcohol use, to HIV risk, and other sexual and reproductive health risks, since people may no longer care what happens to them (12–15). Women are disproportionately affected and a range of countries have reported a gender difference in the experience of mental health effects (6, 16, 17). In Malawi, 48% of women experience some form of IPV, yet data on reported mental health care problems are scarce (18).

IPV is recognised as an enormous public health problem globally and the health sector has a ‘duty of care’ to provide comprehensive health services to survivors of violence, including those relating to its mental health impact (7, 19). However, in practice, health service responses are varied and tend to focus on treating physical injuries and preventing pregnancy and infection; the psychosocial aspects of care are often undervalued and its ‘social emergency’ unrecognised. This has been attributed to lack of health worker capacity to identify and treat mental health problems (20), attitudes which characterise IPV as a private or family matter, and concerns about inappropriate responses that could jeopardise women's safety.

In Malawi, as in many developing countries, responses to IPV are not well integrated into the general health care delivery system but neither are they part of specific mental health, HIV, or family planning counselling services, despite recognition of these as places within the health sector where IPV survivors seek help. In the Malawi Health Sector Strategic Plan (HSSP) (2011–2016), gender-based violence is captured under non-communicable diseases (NCDs) including mental health, alcohol, and trauma, and is included in a list of NCDs that require systems for targeted and routine screening at the primary health care level (21). However, attention has focused on the development at tertiary level of six ‘one stop centres’ aiming to provide comprehensive care and support to survivors of violence (22).

There is a scarcity of evidence as to the effectiveness of interventions aimed at mitigating the mental health effects of violence on both victims and witnesses and at preventing IPV. Context-specific information about individual, community, and health systems responses to IPV is needed to enable the development of locally relevant responses. Very little is known about the types of mental health impact and service responses to mental health needs for survivors of IPV in Malawi. This paper aims to contribute to filling this gap by exploring how the mental health impact of IPV and the capacity of health services for addressing these are perceived by a range of key stakeholders in Malawi; participants included survivors, the general population, and potential/actual service providers, with a focus on health service providers.

We draw on a wider analysis of health sector responses to IPV in Malawi, which included policy analysis and the perceptions of these stakeholders on definitions, causes, and responses to IPV. Our study provides a holistic perspective on the perceived needs for, challenges to, and potential ways forward for developing services to support IPV survivors with mental health issues.

## Methods

### Study design

We employed focus groups discussions and in-depth interviews (IDIs) in this qualitative descriptive study to allow an in-depth analysis of the perceptions of stakeholders in Malawi.

### Study setting

The study was conducted in 2011 in three areas of Blantyre district. Additional data were collected from policy stakeholders and healthcare workers only in Mangochi and Lilongwe districts. Blantyre district has a population of 732,518 and includes both urban and rural areas. Fighting gender-based violence (GBV) has been identified as a priority area for action for gender equity (23). Urban residents are more likely to have access to some services for IPV compared to typical rural areas. The accessibility of these referral points was a key ethical consideration in district selection since the study has the potential to trigger the demand for services for individual participants experiencing violence.

As the main industrial and commercial centre for Malawi, Blantyre's population is ethnically and socio-economically diverse. About 65% of this population lives in unplanned, congested settlements with poor infrastructure and social services (24). Poverty stands at 24% while unemployment stands at 8% (25). About 60% of the district population is below 25 years and the population growth rate is 3.4%, partly due to rural–urban migration (24).

Additional data were collected from policy stakeholders in Lilongwe because they are concentrated here in the country's capital. In addition, Blantyre does not have a Ministry of Health designated district or central hospital because the referral hospital doubles as both. Health care worker (HCW) participants were therefore recruited from Lilongwe central hospital and health facilities under Lilongwe district health office (Mangochi is 150 km from Blantyre) and their sub-district facilities. Furthermore, Lilongwe is the only central hospital among the four that had not yet incorporated the one-stop centre model in its provision of services to survivors of violence.

### Data collection

A total of 28 focus group discussions (FGDs), 3 small group discussions, and 47 individual interviews (in-depth and key informant) were conducted with a range of participants as outlined in Table 1. FGDs with community members were single gender, and conducted in Chichewa. FGDs were employed to help enable participants to share information about the sensitive topics of violence and mental health impact. FGDS can facilitate discussions on taboo topics because less inhibited members of the group may break the ice for shyer participants (26, 27). Group discussions were appropriate for the Malawian context because a culture of individual silence is an obstacle to discussing violence and mental ill-health experiences, but the oral culture is still strong (28) and lends itself to the discussion of sensitive topics in groups (29, 30). Focus group topic guides covered the following main topics: understandings of IPV; perceptions of the magnitude of the problem; the impact of violence (including mental health impact); sources of help-seeking; perceptions of health service responses; and perceptions of the potential role of health services in responding to violence.

**Table 1:** Number of interviews and group discussions

Participants	FGDs (no. of participants)	IIs	KIIs	Small group (no. of participants)	Total no. of participants
Survivors					7
Female		7			
HCWs	10 (88)	14		3 (7)	109
Community members					133
Male	8 (61)				
Female	10 (69)			1 (3)	
Key informants			26	1 (3)	29
Total	28 (218)	21	26	5 (13)	278

### Participant recruitment

Recruitment to FGDs was on the basis of similar age, sex, marital and parental status to create relatively homogenous groups. Participants were recruited to purposively represent both rural and urban areas as part of a maximum variation sample. FGDs were held in villages or townships which required negotiations for community entry with a wide range of gatekeepers. Health surveillance assistants (HSAs) who are community health workers initiated discussions with community gatekeepers. Community leaders, who have administrative and moral obligations for the people in their respective villages, were also involved, and meetings informed them of the purpose. Chiefs, who knew individual households well, approached potential participants through their helpers and HSAs with regard to their potential interest to participate in the study. Interested participants were individually approached by the researchers who gave them detailed information about the study and sought consent for their participation in the study.

IDIs with survivors enabled an in-depth exploration of individual experiences of help-seeking behaviour for IPV and support provided by health services among others (31, 32). Survivors were identified through organisations at which they had sought help (including the police, clinics for the treatment of sexually transmitted infections, community health surveillance assistants and non-governmental organizations) and through snowballing (referral by other participants) (33). Preference was given to survivors who were not currently in abusive relationships, and who were therefore at relatively low risk of retaliation from their abusive partners. However, some participants were in current abusive relationships, requiring additional measures to ensure safety, including secret interview locations and recruitment materials that did not mention IPV.

FGDs with HCWs elicited norms and common experiences regarding identification of and providing care for potential IPV survivors and enabled identification of a range of views, whilst semi-structured interviews with HCWs allowed an exploration of individual providers’ attitudes, perceptions, and experiences. Participant selection sought to ensure homogeneity using professional cadre (nurses, medical assistants, clinical officers, dental and environmental officers), and Village Health Committees (VHCs) as the basis for groups to ensure equal discussion. Very senior staff members were excluded from FGDs. Both were conducted in Chichewa. Key informant interviews (KIIs) were conducted with policy stakeholders, including government ministry staff and donor agencies, as well as GBV service providers, religious institutions, police officers, and other stakeholders; interviews were conducted either in Chichewa or in English.

### Data analysis

Qualitative data were analysed using the ‘framework’ approach (34), assisted by NVIVO 9 software. A thematic coding framework was developed through a process of familiarisation with all the transcripts which enabled the identification of possible categories; this was also informed by *a priori* issues identified in the literature (e.g. perceived burden of IPV, reporting of IPV, health consequences), as well as the aim and objectives of the study. The initial coding framework was revisited and refined through the application of the codes to all the textual data; for example, by identifying new sub-categories and collapsing together linked categories. Matrices were created for each category, with cells for each participant type and sub-category to enable comparing and contrasting of views and linkages between different categories. These were used to identify the two major analytical themes: Malawian perspectives of IPV (that included types, definitions, magnitude, forms, perceived causes of violence, and perspectives on violence and health) and perceptions of IPV services (that included help-seeking and health sector responses). Within this, the initial categories from the framework were re-grouped to reflect the strongest themes emerging across the participant groups through a process of ‘funnelling’ the data. The themes related to mental health consequences were pulled out from this and are used to structure the results section of this paper.

### Ethical approval/consideration

The study was approved by the Liverpool School of Tropical Medicine Research Ethics Committee, the Kamuzu College of Nursing Research and Publications Committee (KCNRPC), and the Malawi College of Medicine Research and Ethics Committee (COMREC). Permission to conduct the study was also obtained from the Hospital Director of Queen Elizabeth Central Hospital, Blantyre District Health Office, in charges of respective health centres, service organisations, and community leaders in various communities and individual participants. A range of quality assurance measures were undertaken and data were triangulated across methods and interviewees. All participants gave full informed consent to participate in the study.

## Results

The research strategy employed in the study elicited multiple perspectives on: IPV and its mental health impact; help-seeking strategies perceived by the community and employed by survivors; the adequacy of services provided by healthcare providers for mental health impacts; and the challenges faced by healthcare providers in meeting the needs of the survivors. The analysis presented in this paper focuses on perceptions and experiences of violence against women, although it includes the perceptions of male community members and HCWs in FGDs concerning the mental health impacts of IPV and health service responses.

### Participant understandings of violence

The concept of violence (*nkhanza*) was perceived by participants to cover a wide spectrum of abusive behaviours. Against a woman, these range from marital rape, sexual assault, and physical violence to neglect, controlling behaviour, too frequent demands for sex, having concurrent relationships (with attendant HIV risk), and unequal division of love in polygamous relationships. A link between alcohol abuse and violence perpetration was identified by both women and men. Although forced sex within marriage was acknowledged in both female and male FGDs and interviews, many participants did not interpret this as rape.

Many participants stressed the need for an inclusive definition of IPV that goes beyond physical and sexual violence. A general consensus was that violence is wrong and violates an individual's rights, but also that is expected in marital relationships:

I used to treat violence as part of the normal married life … because people have said that such things sometimes do happen in marriage. (Female survivor, interview)

The level of IPV was perceived as high by all participants and was often seen as increasing:

To say the truth violence here is too much. The kind of violence which we meet in the house … are very big such that if we are to expose it here, can make everybody's head confused … (Elderly men, urban FGD)

### The perceived mental health impacts of IPV

Both women and men in FGDs and interviews perceived adverse psychological impacts of violence as common, describing depression, anxiety, fear, and suicidal ideas and attempts. Many participants made no distinction between IPV and its mental health impact, describing the lack of ‘peace of mind’ as violence:

Violence means depriving the other of peace particularly peace of mind because peace of mind contributes to good health. Therefore I can say that deprivation of peace or beating is some form of violence. (Female survivor, interview)

Constant anxiety was commonly described as a consequence of living with IPV by women in FGDs. Those women with controlling husbands talked about their attempts to anticipate and avoid negative reactions to daily events:

You are always anxious and full of fear, thinking how he is going to receive me when I arrive home, what I am going to experience, how things are going to be? (Older women, rural FGD)

Controlling husbands who restricted their wives’ social interactions also sometimes deprived them of an opportunity to reduce their stress by laughing and joking with friends. Anxiety was sometimes said to manifest externally by talking to oneself out loud. This state of anxiety was compared by several participants to imprisonment or slavery:

Even if your friends are joking, laughing you don't because you are worried, thinking about the problems that are surrounding you. You live unhappily all the time. You are like a slave as such you are not free because of violence. (Older women, rural FGD)

Health was perceived in a holistic way by many, with mental health impacts often described as leading to physical ill-health. For example, both women and men in FGDs discussed how anxiety and ‘stress’ or ‘thinking too much’ could lead to loss of appetite and weight loss, as well as physical illness:

Stress may result in premature death because of thinking too much which may result in complications resulting into frequent illness and death. (Older women, urban FGD)

Both women and men talked about people experiencing IPV, feeling suicidal, and in some cases attempting suicide. Alcohol abuse was also seen by some as a coping mechanism by those experiencing IPV:

You find that a person who was not drinking alcohol becomes addicted to alcohol or else smoking “chamba” (cannabis) for them to forget worries they encounter. (Young men, urban FGD)

Both female and male participants described the psychological impact of witnessing violence in the home on children, including anxiety and feeling neglected. Young participants described witnessing parental violence as shameful, leading to a sense of hopelessness, threatening their feelings of security in the home, and their social and educational development:

It's very shameful to watch your parents fighting every day and for people to watch them fighting every day. The person that feels more ashamed is the one that is not involved in the fight … Many children have gone astray because of such situations. (Young women, urban FGD)

Even at school you don't function well because you are occupied with the thought about the violence that you have witnessed. (Young women, urban FGD)

Some HCWs expressed the view that experiencing IPV was an underling factor for many women presenting with psychiatric problems such as ‘hysteria’, fainting attacks, depression, and minor illnesses of unknown origin.

### Community responses to IPV: help-seeking behaviours and experiences

All participants described a prevailing culture of silence surrounding marital issues and expressed perceptions of marriage as endurance:

We are taught that marriage is endurance. This means hiding whatever is happening in the home. (Older women, rural FGD)

It was generally perceived that survivors of IPV endure violence for a long time before it is made public, and this was the case amongst those survivors interviewed. Help-seeking was generally described as initiated either due to escalating severity or long duration of violence:

Most of them who come here will say that things have reached beyond endurance … they are fearing that they might die, meaning that she has lived with that for quite a long time but she was silent. (Female HCW I, hospital interview)

When help was sought, the majority used informal sources, including family, friends, neighbours, traditional marriage counsellors, and religious and traditional leaders. A minority sought more formal services such as health services, police, and non-governmental organisations. Among informal sources, the key role played by appointed marriage ‘guardians’ or *nkhoswe* within the traditional marriage system was widely stressed in all the FGDs and interviews. The *nkhoswe* are expected to resolve conflict, build marriages, and promote behaviour change by perpetrators. However, most participants discussed limitations to their effectiveness due to lack of skills in counselling, burnout in dealing with repeated violence, allegiance to one side of the family, and their tendency to downplay violence in their efforts to promote resolution.

With regard to formal services, the police and health services were seen as the main sources of help in FGDs and interviews; their help is sought particularly in response to severe physical violence. Survivors interviewed also sought help from community based organization working on violence. In general, the police were seen as responsive, but their focus was also on mediation and conflict resolution, including persuading offenders to change their behaviour. Both community members and police interviewees commonly saw police action and legal proceedings against perpetrators as conditional on the survivor deciding to end the relationship. Many participants reported this as a challenge for women to seek help or disclose violence, due to their financial dependence on the perpetrator. Health services were generally sought in response to physical injury requiring medical care and, less commonly, rape. Whilst some participants expressed a hope that health workers would talk to them and support them, most feared judgemental attitudes.

### Health service and other sector responses to mental health impact of IPV

Health workers themselves as well as health policy stakeholders acknowledged that in general they limited their responses to IPV to treating physical health and did not encourage disclosure or attempt to address mental health or emotional well-being. Both providers and policy makers attributed this to negative attitudes of some providers towards IPV survivors, a perceived lack of health sector mandate to address psychosocial aspects of health, lack of skills for counselling due to lack of training, lack of time with patients, and lack of referral options. One health worker interviewed denied the existence of IPV and encouraged women to resolve the issue themselves:

I don't know whether I am wrong but in this world there is no violence. It exists because people have labelled misunderstandings between two partners as violence … Every patient that comes with police report, when I start questioning them, the story they narrate is very small. I tell them sister (because the majority of the patients that I see here are women) …, why can't you discuss; not necessarily going to the marriage advocate but discuss in your own house. (Male HCW, rural health centre interview)

Some HCWs saw addressing psychosocial needs as beyond their mandate:

It can be helpful but it is a big challenge because sometimes we will be going deeper into places we are not supposed to go as health care providers. The expectation of the community is that our role is to treat people with medication. (Female HCW, hospital interview)

Most commonly health workers recognised that survivors of violence needed psychosocial support, which was generally viewed in terms of ‘good’ or ‘proper’ ‘counselling’:

We need counsellors at the hospital. Most of the clients that we see at the hospital do not suffer from infections but rather psychological issues. I feel we could have eliminated a lot of problems in the health services if counselling was to be done. (Mixed, urban health centre FGD)

In most interviews, HCWs asserted that they do provide counselling to survivors of violence. However, many seemed uncertain about what counselling was; interpretations included conflict resolution, education, information, and advice giving. Some HCWs admitted that they were better at education and advice giving than person-centred counselling. One of the providers with psychiatric training commented:

I am talking from experience that these patients are not counselled. We can say that people mistake counselling for advice giving. What we do better is education and advice giving which doesn't assist the patient at all. (Female, hospital small group discussion)

Many participants felt that training curricula based on the bio-medical model of health made them less prepared to deal with psychosocial issues:

The education system didn't look at it to be something big to be brought into the curriculum … our health system has mostly concentrated on physical illness leaving aside other issues like psychological or social. This is why it hasn't been included but I wish it was included. (Female HCW, hospital interview)

Health workers reported that in pre-service training, GBV was listed as a ‘self-study topic’ and mentioned in passing as either a consequence or determinant of a ‘major health problem’, and little interest was shown by either lecturers or students.

Health workers also emphasised that their burden of work due to staff shortages limited the time they had to interact in any depth with patients:

We have a lot of work … and also because of lack of skill … This is why we deal with the physical problem only and leave the psychological or just little bit of it and send the patient back home. (Mixed, rural community hospital FGD)

At one of the health centres, providers explained that under-reporting and consequent low case rates have further impacted on their skills and competency through lack of opportunities to gain confidence in dealing with cases.

HCWs suggested a range of ways forward for improving counselling services to survivors of violence. Whilst some proposed training in counselling skills, others suggested referral to psychiatric nurses and or other organisations such as social services for counselling. Concern was expressed about adding counselling to an already high workload.

Maybe if the government can do something about it; may be recruiting special counsellors for some of these issues so that we can refer these clients to them … but expecting the same nurse to see patients, attend antenatal and work in labour ward, the same doctor should attend to outpatient clients and even have spare time for counselling people it is very difficult. (Mixed, urban health centre FGD)

### Policy stakeholders and donors responses

KIIs with policy stakeholders, police, and NGOs also revealed a perceived lack of capacity for psychosocial support, including counselling, across other governmental and non-governmental services that deal with survivors of IPV. Those who were aware of the restructuring of the Ministry of Health felt that the new unit that combined violence and mental health provided an opportunity to address this. Interviews with community police officers identified counselling skills as an area where they required training. Similarly, most interviewees from non-governmental organisations acknowledged that they are not confident with the counselling services they offer to survivors.

Most organizations work on assumptions because there is lack of skills in most stakeholders. There is need for a fully-fledged programme to deal with trauma. (Male GBV service provider, interview)

The capacity of social workers to provide counselling was also doubted by other service providers. One key informant summed up the discussion and revealed uncertainty as to who has the responsibility for counselling survivors of violence:

There is a lot of psychological damage which I don't think our hospitals are addressing. … I don't think we have such a service so I don't know whose job it is? Is it we NGOs or the hospital? As for us we can claim that we do counselling. The truth is we don't … This is where we have a … short fall or a gap in our system. Even in the police we need trained counsellors, in the hospital we need trained counsellors, even us as NGOs we need trained counsellors to be handling these issues but now it's just a matter of trial and error. (Female, NGO interview)

Interviews with donors, as policy stakeholders working in violence-related areas, revealed that they were both involved with the justice and community organisations and with different departments of the Ministry of Health, yet without proper coordination. For example, the nursing department coordinated the prevention of child maltreatment programme and the HIV/AIDS unit worked on the one stop centres. Whilst HCWs articulated their mandate in terms of treating or responding to violence, the health donors were focused on violence reduction as this quote illustrates:

Our primary focus is prevention of violence using the public health Approach. We emphasise on prevention rather than reacting to incidences. Reacting to violence which has already occurred is an expensive intervention although prevention may have a long term health impact. (Male health developmental partner, KII)

Another health development partner indicated that it had occurred to them for the first time during the interview that health services have the potential for working in violence prevention

We are on the side of support. I don't think we have been involved in any area of prevention through primary health care. This should be an interesting area to look at. Counselling couples who are going to have a baby. Often violence occurs during pregnancy isn't it? Actually seeing you sitting there this is the first time that it has occurred that there is a gap that I don't think is covered by anyone. (Female health developmental partner, KII)

## Discussion

Our findings have shown a wide range in types of perceived mental health impact of IPV among women in Malawi, where individual and community-level tolerance of violence exposes both resilience and vulnerability. Anxiety, low self-esteem, and depression were commonly described negative consequences, but survivors also mentioned suicidal ideation and suicide. Alcohol use was seen both as a consequence and as a trigger of violence. We report common help-seeking behaviours and the strong desire for support that is perceived to be absent or inadequate by community members and service providers alike. Cultural pressures that favour conflict resolution mean that ‘enduring’ marriage was viewed as a common experience. On the one hand this was linked to family mechanisms for support in the form of traditional marriage counsellors, potentially building resilience. On the other, the barriers to effectively resolving or escaping violent relationships exacerbated feelings of isolation, anxiety, and depression. HCWs and other sectors expressed the need for and willingness to engage more through basic counselling support but felt they lacked training, skills, or onward referral points within the system, making them reluctant to tackle mental health issues related to violence in practice.

While there are data from Malawi confirming the link between IPV and depression among pregnant women (35), evidence for effective interventions that prevent violence is limited (30), and there have been no studies aimed at methods for mitigating mental health consequences. The effectiveness of mental health interventions are different in the context of violence, where the cycle may start in childhood due to witnessing violence (36) and/or the external mental ill-health trigger of violence may be ongoing. Intervention may therefore be required to prevent continued violence in order to effectively address its mental health impact. There is some international evidence to support a range of counselling approaches for secondary and tertiary prevention of violence (37) although there is debate about whether this is best provided to individuals (to either or both partners), couples, or groups. Six sessions of counselling for women seeking IPV services was found to be effective in decreasing HIV risk in South Africa (38), and in Namibia, brief motivational interviewing was successfully used in alcohol reduction programmes, although the impact on IPV was not formally evaluated (39).

Data from high-income countries suggest that therapy approaches for couples, including conjoint couples therapy to reduce IPV and alcohol dependence, have an impact in reducing violence where couples stay together (40). There is no direct evidence from low-income countries, but marital communication skills development was found to be an essential mechanism for successfully involving men in couples’ contraceptive decision-making and increased use of contraceptives in Malawi (41). Couples HIV Testing and Counselling (CHTC) combined with IPV screening interventions that address gender inequalities have also improved outcomes following interventions for the prevention of mother to child transmission of HIV (42–44). The strong pressure for couples to stay together may provide incentives for some couples to enter therapy together; however, counselling those with a desire for resolution would have to be accompanied by screening mechanisms that ensured only appropriate couples, where both partners were likely to benefit are included (40). However, counselling provides only one component of an effective response to mental health issues associated with IPV and as such needs to be located within a wider set of options for legal, social, and financial support for survivors who decide to exit violent relationships and bring perpetrators to justice (45).

Utilisation of healthcare services as a common source of formal help-seeking implies the need for basic capacity in ‘first line’ support, identifying survivors, facilitating diclosure, and making appropriate referrals at all levels (19). Promoting empathetic health care provision is challenging. More awareness training and sensitisation could help, especially if courses focus on women's needs and strengths and how health providers can validate these and contribute to a longer term process of change for victims of violence. Clear guidance on how to record history of abuse, ask questions sensitively, and validate experiences is also important together with training on good communication skills such as listening and being empathetic.

An opportunity for effective integration of secondary violence prevention and mental health services exists within the Department of Non-communicable Diseases in the Ministry of Health in Malawi, which is responsible for both. There is recognition at the policy level that mental health problems and IPV are interlinked because they share common risk factors including alcohol and drug use. Specialist interventions that are geared towards addressing mental health, alcohol/drug abuse, and IPV and their intersections are needed (46). These may include screening for IPV in mental health services, screening for mental health problems in IPV services, and referral to individual, couples, and/or group counselling sessions in addition to legal, financial, and social services. Wider structural interventions for primary prevention of violence are ultimately required to impact on the burden of mental ill-health resulting from violence at the community level.

Our study has a number of limitations. First, we focused our paper on women. Previous data from the Malawi Demographic Health Survey (18) and from trauma registries (47) indicate that women experience a high prevalence of IPV. The high levels of mental health consequences among women are validated by previous studies (35) and gendered power relations create a qualitative difference between IPV against women and against men. Nevertheless male participants also describe experiencing violence and mental ill health and turning to drugs and alcohol as a result of marital disharmony, which in turn may perpetuate cycles of violence perpetration. Second, this study was not designed to focus specifically on the mental health impact of violence, which was considered part of a wider health and social impact. Exploration of the views of the participants did not therefore focus on their views of the most effective approaches to reduce mental health impacts and, in particular, perceptions of survivors and community members about the potential effectiveness of counselling approaches were not elicited. Third, the individual experiences documented in this study are limited to narratives from a small number of individual survivors selected through referral from support organisations and snowball sampling. As a result, most had experienced particularly severe forms of violence. We aimed to complement this through the use of community FGDs to elicit community norms. A range of different voices were purposively selected including younger and older women and men. However, the scale and complexity of the data as well as the difficulties in distinguishing between rural, peri-urban, and urban settings in Blantyre limited opportunities to clearly distinguish the influence of these different factors on their perspectives and experience of help-seeking.

## Conclusion

There is widespread recognition of the mental health impact of IPV on women in Malawi and the public health burden this poses, but formal care seeking was thought to be impeded by social pressures to resolve conflict, and fear of judgemental attitudes. Providers felt inadequately prepared to handle the psychosocial and mental health consequences of IPV, and there is currently a limited capacity to support affected individuals at the community or the health sector level. Participants highlighted potential entry points to health services as well as local and national opportunities for interventions that are culturally appropriate and are built on local structures and resilience. Violence prevention efforts could take advantage of community resilience, local systems, as well as existing funding structures. These efforts may incorporate community-level interventions with children and adolescents; working with marriage counsellors on building healthy relationships; and focus on couples’ counselling through existing HIV services. These would both help to move the IPV agenda in Malawi from documenting violence and its impact to one of targeted prevention efforts that mitigate mental health consequences.

## References

[1] Krug EG, Mercy JA, Dahlberg LL, Zwi AB (2002). The world report on violence and health. Lancet.

[2] Devries KM, Mak JY, Bacchus LJ, Child JC, Falder G, Petzold M (2013). Intimate partner violence and incident depressive symptoms and suicide attempts: a systematic review of longitudinal studies. PLoS Med.

[3] Rosen D, Seng J, Tolman R, Mallinger G (2007). Intimate partner violence, depression, and posttraumatic stress disorder as additional predictors of low birth weight infants among low-income mothers. J Interpers Violence.

[4] Ellsberg M, Jansen HA, Heise L, Watts CH, Garcia-Moreno C (2008). Intimate partner violence and women's physical and mental health in the WHO multi-country study on women's health and domestic violence: an observational study. Lancet.

[5] Humphreys C, Thiara R (2003). Mental health and domestic violence: ‘I call it symptoms of sbuse’. Br J Soc Work.

[6] Jina R, Jewkes R, Hoffman S, Dunkle KL, Nduna M, Shai NJ (2012). Adverse mental health outcomes associated with emotional abuse in young rural South African women: a cross-sectional study. J Interpers Violence.

[7] Devries K, Watts C, Yoshihama M, Kiss L, Schraiber LB, Deyessa N (2011). WHO Multi-Country Study Team Violence against women is strongly associated with suicide attempts: evidence from the WHO multi-country study on women’s health and domestic violence against women. Soc Sci Med.

[8] Bengtsson-Tops A, Tops D (2007). Self-reported consequences and needs for support associated with abuse in female users of psychiatric care. Int J Ment Health Nurs.

[9] Tadegge AD (2008). The mental health consequences of intimate partner violence against women in Agaro Town, southwest Ethiopia. Trop Doctor.

[10] Gelaye B, Arnold D, Williams MA, Goshu M, Berhane Y (2009). Depressive symptoms among female college students experiencing gender-based violence in Awassa, Ethiopia. J Interpers Violence.

[11] Deyessa N, Berhane Y, Alem A, Ellsberg M, Emmelin M, Hogberg U (2009). Intimate partner violence and depression among women in rural Ethiopia: a cross-sectional study. Clin Pract Epidemiol Ment Health.

[12] Rehm J, Shield KD, Joharchi N, Shuper PA (2012). Alcohol consumption and the intention to engage in unprotected sex: systematic review and meta-analysis of experimental studies. Addiction.

[13] Li Y, Marshall CM, Rees HC, Nunez A, Ezeanolue E, Ehiri J (2014). Intimate partner violence and HIV infection among women: a systematic review and meta-analysis. JIAS.

[14] Cagney H (2014). Intimate partner violence and HIV: unwelcome accomplices. Lancet.

[15] Limaye RJ, Rutkow L, Rimal RN, Jernigan DH (2014). Informal alcohol in Malawi: stakeholder perceptions and policy recommendations. J Public Health Policy.

[16] Romito P, Grassi M (2007). Does violence affect one gender more than the other? The mental health impact of violence among male and female university students. Soc Sci Med.

[17] Tran T, Tran T, Wynter K, Fisher J (2012). Interactions among alcohol dependence, perinatal common mental disorders and violence in couples in rural Vietnam: a cross-sectional study using structural equation modeling. BMC Psychiatry.

[18] National Statistical Office (NSO) & MACRO (2005). Malawi Demographic and Health Survey 2004.

[19] WHO (2013). Responding to intimate partner violence and sexual violence against women: WHO clinical and policy guidelines.

[20] Kauye F (2008). Management of mental health services in Malawi. Int Psychiatry.

[21] GOM (2011). Malawi health sector strategic plan 2011–2016 moving towards equity and quality.

[22] MOH (2012). Guidelines for Provision of Comprehensive services for survivors of physical and sexual violence at Health Facilities in Malawi (One-Stop Centres).

[23] Tim Mercer and MCI (2010). MCI SOCIAL SECTOR WORKING PAPER SERIES Gender Needs Assessment for Blantyre City, Malawi;. http://mci.ei.columbia.edu.

[24] United Nations Human Settlements Programme Malawi (2011). http://www.unmalawi.org/agencies/unhabitat.html.

[25] Bandawe LR (2006). Managing the HIV and AIDS pandemic at the local level: experience from the Blantyre City Assembly;. http://mirror.unhabitat.org/downloads/docs/4058_97151_Blantyre.pdf.

[26] Krueger RA, Casey MA (2000). Focus Group A practical guide for applied reseach.

[27] Kitzinger J, Barbour RS (1999). Developing focus group research: politics, theory, and practice.

[28] Tough A (2012). Oral culture, written records and understanding the twentieth-century colonial archive. The significance of understanding from within. Arch Sci.

[29] Theobald S, Nyirenda L, Tulloch O, Makwiza I, Soonthorndhada A, Tolhurst R (2011). Sharing experiences and dilemmas of conducting focus group discussions on HIV and tuberculosis in resource-poor settings. Int Health.

[30] Mkandawire-Valhmu L, Stevens PE (2010). The critical value of focus group discussions in research with women living with HIV in Malawi. Qual Health Res.

[31] Mason J (1998). Qualitatieve researching.

[32] Legard R, Keegan J, Ward K, Ritchie J, Lewis J (2003). Qualitative research practice: a guide for social science students and researchers.

[33] Pole C, Lampard R (2002). Practical social investigation: qualitative and quantitative research methods in social research.

[34] Ritchie J, Lewis J (2003). Qualitative research practice: a guide for social science students and researchers.

[35] Stewart R, Umar E, Tomenson B, Creed F (2014). A cross-sectional study of antenatal depression and associated factors in Malawi. Arch Women's Ment Health.

[36] Heise L (1998). Violence against women: an integrated, ecological framework. Violence Against Women.

[37] Wolfe DA, Jaffe PG (2003). Prevention of domestic violence and sexual assault. VAWnet, a project of the National Resource Center on Domestic Violence/Pennsylvania Coalition Against Domestic Violence.

[38] Sikkema K, Neufeld S, Hansen N, Mohlahlane R, Van Rensburg M, Watt M (2010). Integrating HIV prevention into services for abused women in South Africa. AIDS Behav.

[39] Fritz K, Gregowski A, Glenshaw M, DeLuca N, Hurwitz E, Cornman H (2012). Addressing the impact of alcohol on the prevention, care, and treatment of HIV in Southern and Eastern Africa: research, programming, and next steps-report on a PEPFAR technical consultation. USAID's AIDS Support and Technical Assistance Resources.

[40] McCollum EE, Stith SM (2008). Couples treatment for interpersonal violence: a review of outcome research literature and current clinical practices. Violence Vict.

[41] Hartmann M, Gilles K, Shattuck D, Kerner B, Guest G (2012). Changes in couples’ communication as a result of a male-involvement family planning intervention. J Health Commun.

[42] Becker S, Mlay R, Schwandt H, Lyamuya E (2010). Comparing couples’ and individual voluntary counseling and testing for HIV at antenatal clinics in Tanzania: a randomized trial. AIDS Behav.

[43] Semrau K, Kuhn L, Vwalika C, Kasonde P, Sinkala M, Kankasa C (2005). Women in couples antenatal HIV counseling and testing are not more likely to report adverse social events. AIDS.

[44] Farquhar C, Kiarie JN, Richardson BA, Kabura MN, John FN, Nduati RW (2004). Antenatal couple counseling increases uptake of interventions to prevent HIV-1 transmission. J Acquir Immune Defic Syndr.

[45] Mercy JA, Rosenberg ML, Powell KE, Broome CV, Roper WL (1993). Public health policy for preventing violence. Health Aff.

[46] Stith SM, McCollum E, Amanor-Boadu Y, Smith D (2012). Systemic perspectives on intimate partner violence treatment. J Marital Ther.

[47] Kiser M, Escamilla V, Samuel J, Eichelberger K, Mkwaila J, Cairns B (2013). Sex differences in interpersonal violence in Malawi: analysis of a hospital-based trauma registry. World J Surg.

